# The Particle Radiobiology of Multipotent Mesenchymal Stromal Cells: A Key to Mitigating Radiation-Induced Tissue Toxicities in Cancer Treatment and Beyond?

**DOI:** 10.3389/fonc.2021.616831

**Published:** 2021-04-12

**Authors:** Alexander Rühle, Anca-Ligia Grosu, Nils H. Nicolay

**Affiliations:** ^1^ Department of Radiation Oncology, University of Freiburg - Medical Center, Freiburg, Germany; ^2^ German Cancer Consortium (DKTK) Partner Site Freiburg, German Cancer Research Center (dkfz), Heidelberg, Germany; ^3^ Department of Molecular Radiation Oncology, German Cancer Research Center (dkfz), Heidelberg, Germany

**Keywords:** stem cell therapy, normal tissue toxicities, radiotherapy, particle irradiation, mesenchymal stem cells, mesenchymal stromal cells, space irradiation, radiation accidents

## Abstract

Mesenchymal stromal cells (MSCs) comprise a heterogeneous population of multipotent stromal cells that have gained attention for the treatment of irradiation-induced normal tissue toxicities due to their regenerative abilities. As the vast majority of studies focused on the effects of MSCs for photon irradiation-induced toxicities, little is known about the regenerative abilities of MSCs for particle irradiation-induced tissue damage or the effects of particle irradiation on the stem cell characteristics of MSCs themselves. MSC-based therapies may help treat particle irradiation-related tissue lesions in the context of cancer radiotherapy. As the number of clinical proton therapy centers is increasing, there is a need to decidedly investigate MSC-based treatments for particle irradiation-induced sequelae. Furthermore, therapies with MSCs or MSC-derived exosomes may also become a useful tool for manned space exploration or after radiation accidents and nuclear terrorism. However, such treatments require an in-depth knowledge about the effects of particle radiation on MSCs and the effects of MSCs on particle radiation-injured tissues. Here, the existing body of evidence regarding the particle radiobiology of MSCs as well as regarding MSC-based treatments for some typical particle irradiation-induced toxicities is presented and critically discussed.

## Introduction

Mesenchymal stromal cells (MSCs) were first isolated from the human bone marrow by Friedenstein and colleagues in the late 1960s (1, 2), but have since been described in various other tissue types such as adipose and glandular tissues, brain and umbilical cord (3–6) and many other organs. As MSCs are a heterogeneous population that can only be characterized by combining molecular and functional traits, the International Society for Cellular Therapy (ISCT) proposed minimal defining criteria for MSCs in order to enhance comparability between the various studies: MSCs are required to adhere to plastic surfaces, exhibit a pattern of positive and (absent) negative surface markers and possess the ability to differentiate along the adipogenic, osteogenic and chondrogenic lineages (7). Currently, MSC-based treatments hold approval for several indications including graft-versus-host disease, Crohn’s-related enterocutaneous fistular disease, bone regeneration, osteoarthritis and cartilage repair (8–11). MSCs possess several characteristics that make them an attractive cell type for cell-based treatments: MSCs can be conveniently isolated and expanded; they are immune-privileged cells, therefore not requiring immunosuppression prior to application, and exhibit the ability to migrate to damaged tissues (12–15). Meta-analyses with more than 1000 patients showed the general safety of MSC-based treatments and did not reveal any severe adverse effects (16). As MSCs also home to tissues damaged by ionizing radiation, these cells came into focus as potential treatments for radiation-induced toxicities, for instance, radiation mucositis, pulmonary fibrosis and enteritis (17–20).

In the context of treating radiation-induced tissue injuries, the MESRIX trial provided a major step towards the routine usage of MSCs in radiation oncology. This randomized, placebo-controlled, double-blinded phase I/II study evaluated for the first time the efficacy of an MSC application for radiotherapy-induced tissue damage (21): Autologous adipose tissue-derived MSCs were transplanted into the submandibular salivary glands of patients with severe radiotherapy-related xerostomia at three or more years after treatment. Upon MSC administration, salivary flow rates were found significantly increased compared to the placebo group, and consistently, xerostomia symptoms decreased in MSC-treated but not in placebo-treated patients. Based on these encouraging results, a follow-up trial (NCT03874572) aims to validate these findings also for allogenic MSCs.

So far, most *in vitro* and *in vivo* studies have focused on the impact of photon irradiation on MSCs, and little is known about the particle radiobiology of these multipotent cells, which is a crucial step towards the usage of MSC-based therapies for particle radiation-associated tissue damage, e.g. after particle radiotherapy or during manned deep space flight (22–24).

## Material and Methods

The databases PubMed, Google Scholar and Web of Science were screened for studies investigating the impact of MSC-based treatments for particle irradiation-induced toxicities as well as the influence of particle irradiation on MSCs by using the search terms *mesenchymal stem cells*/*mesenchymal stromal cells* in combination with *protons*, *proton radiotherapy*, *helium ions*, *alpha particles*, *carbon ions*, *carbon ion radiotherapy*, *oxygen ions*, *iron ions*, *heavy ions* and *heavy ion radiotherapy*, respectively.

## Application of MSCs for the Attenuation of Typical Toxicities After Particle Radiation

### Particle Radiotherapy in Cancer Treatment

Currently, heavy ion radiotherapy most commonly employs ^12^C carbon ions for patient treatment. Clinical feasibility and benefits of carbon ion radiotherapy have been studied in various malignancies such as intracranial tumors, head-and-neck cancers, lung cancer, gastrointestinal malignancies, prostate cancer, sarcomas, gynecological cancers and pediatric malignancies (25).

#### Head-and-Neck Tumors

Particle radiotherapy has been shown to be an effective treatment for salivary gland, nasopharyngeal and sinunasal carcinomas (25–29); however, bone- and mucosa-related toxicities are common, and no causative treatments have been licensed to date for these radiation-induced lesions.

Radiation-related mucositis is a common acute normal tissue toxicity and affects most patients receiving radiotherapy for head-and-neck malignancies. It considerably worsens patients’ quality of life, increases the risk for malnutrition and infection and can therefore result in treatment interruptions that may in turn deteriorate oncological outcomes. The incidence of severe mucositis after proton irradiation for head-and-neck squamous cell carcinomas ranges between 40 and 79% (30). The impact of both bone marrow- and adipose tissue-derived MSCs on radiation-induced mucositis has been examined in several preclinical studies (19, 31–33). In the study of Maria and colleagues, MSCs significantly attenuated radiation-induced oral mucositis in mice, as evident by reduced ulcer duration and ulcer size, leading to increased weight as well as improved hydration and nutritional status (31). Osteoradionecrosis constitutes another rare but severe chronic toxicity after head-and-neck radiotherapy and is characterized by chronically exposed bone structures that fail to heal within 90 days after irradiation. In the head-and-neck region, osteoradionecrosis commonly affects the mandibular bone, and the incidence of mandibular osteoradionecrosis is reported to be less than 10% in most studies after intensity-modulated radiotherapy, although the variation of incidence in the literature is wide (34). The pathophysiology of osteoradionecrosis is complex and is driven by chronic inflammation, hypovascularity, hypoxia and hypocellularity (35). Treatment aims at alleviating symptoms and may comprise antibiotic treatment, surgical debridement, reconstructive surgery and hyperbaric oxygen; however, management of mandibular osteoradionecrosis remains a clinical challenge (36). Although the incidence of mandibular or maxillary osteoradionecrosis may be lower after carbon ion radiotherapy compared to photon radiotherapy, there are several case reports describing severe cases of osteoradionecrosis after carbon ion treatment (37, 38). In a retrospective analysis, 3 of 63 patients developed grade 3 osteoradionecrosis after carbon ion radiotherapy of head-and-neck malignancies (39). Stem-cell based treatments using MSCs obtained from the bone marrow, adipose tissue or tonsils have been examined for osteoradionecrosis after photon irradiation in four *in vivo* studies and in two clinical case reports (40–46).

While it is conceivable that MSCs may exert their beneficial effects regarding immunomodulation and replacement of functional cells also after carbon ion irradiation, confirmative data for the treatment of particle radiation-induced mucositis and osteoradionecrosis are still lacking.

#### Prostate Cancer

In a *post-hoc* analysis including more than 1000 patients with prostate cancer receiving carbon ion radiotherapy within prospective phase II trials, the 10‐year rates of moderate-to-severe gastrointestinal and genitourinary toxicities were found to be 1.7% and 11.7%, respectively (47). Analyses of the SEER database comparing photon and proton radiotherapy for prostate cancer observed an increased risk for rectal bleeding in patients treated with particle radiation (48). MSCs have been successfully investigated for radiation-induced proctitis, cystitis and fistulas. For instance, four patients received allogeneic bone marrow-derived MSCs for hemorrhagic radiation-induced fistulizing colitis after a radiation oncology accident at a public hospital in France, in which prostate cancer patients were overdosed by up to 30% (49). While two patients exhibited a significant response regarding pain and hemorrhage, another patient experienced a relapse after 6 months that was responsive to a second MSC administration. Considering these encouraging results, a prospective phase II trial is currently evaluating the impact of MSC-based treatments for late severe gastrointestinal complications such as proctitis and cystitis (PRISME trial, NCT02814864). However, similar to the other discussed late sequelae here, there are no studies available that exclusively investigate MSC-based therapies after proton or carbon ion radiotherapy.

### MSC Administration as a Treatment Against Acute Radiation Syndrome

The acute radiation syndrome (ARS) represents a complex clinical situation, consisting of the hematopoietic syndrome, the gastrointestinal syndrome and (only clinically relevant for doses exceeding 10 Gy) the cerebrovascular syndrome (50). ARS regularly occurs after radiation exposure of the whole body with doses exceeding 0.5-1 Gy over a short time period. Currently, the standard of care for ARS consists of mainly symptomatic measures, e.g. intravenous hydration, antiemetic and analgesics medication, antibiotic treatment, blood transfusions, application of hematopoietic growth factors and rarely stem cell transplantation. Depending on the extent of medical interventions, the LD_50/60_ (dose that kills 50% of the population within the first 60 days) ranges between 2.5 and 5 Gy. There are several scenarios in which a particle radiation-induced ARS may occur, e.g. during radiation accidents, nuclear terrorism or warfare and in deep space. The rising number of centers providing particle radiotherapy as well as the increasing usage of nuclear technology for medical purposes, industrial procedures and military functions emphasizes the importance of effective medical countermeasures to treat particle radiation-induced ARS.

Animal studies have shown beneficial effects of MSC-based treatments (including both bone marrow MSCs and MSC-derived exosomes) on the hematopoietic system in lethally irradiated mice (51–54). There are also several case reports showing both the feasibility and efficacy of MSC-based therapies for the treatment of tissue damage caused by radiation accidents (55–58). However, no studies have yet been reported that described the impact of MSC-based therapies on particle irradiation-induced ARS. MSCs from different tissues of origin (bone marrow and umbilical cord) have successfully been investigated for ARS treatment (51, 59). Besides its relevance for unintended radiation accidents, stem cell-based treatments including both MSCs and hematopoietic stem cells are discussed also in the case of nuclear terrorism (60). In this context, the United States Army Medical Research Institute of Chemical Defense (USAMRICD) has established production methods in order to have MSC-based treatments available for ARS (61).

## Responses of MSCs to Particle Radiation Exposure

For photon radiotherapy, it has been demonstrated that MSCs from different tissues of origin (e.g. bone marrow, adipose tissue, umbilical cord) are relatively radioresistant and maintain their stem cell traits even after high radiation doses (23, 62, 63). A high anti-oxidative capacity, an effective DNA double-strand break repair, low levels of pro-apoptotic proteins (e.g. Bim and Puma) accompanied by high levels of anti-apoptotic proteins (e.g. Bcl-2 and Bcl-XL) have been reported to contribute to photon radioresistance of these multipotent stromal cells (64, 65). However, only limited data are available on the particle radiobiology of MSCs, and it remains unclear if the photon radioresistance can be extrapolated to radiation with protons or heavier particles. To date, proton radiation has not yet been systematically studied regarding the MSC radiobiology as it is believed to biologically resemble photon radiation. However, there are limited data available on the radiation effects of ^12^C and heavier particles such as ^56^Fe on MSCs.

### Cellular Survival After Particle Irradiation

Work by our group demonstrated a relatively radioresistant phenotype of bone marrow-derived MSCs after ^12^C particle radiation (**Table 1**). The relative biological effectiveness (RBE) calculated at 10% clonogenic survival in bone marrow-derived MSCs ranged between 2.0 and 3.1 compared to photon radiation, underpinning the known heterogeneity of MSCs (69). In line with a radioresistant phenotype after particle radiation, apoptosis rates in bone marrow-MSCs remained low even after exposure to 4 Gy of ^12^C irradiation. However, no data are available for the influence of protracted courses of low-dose ^12^C radiotherapy on MSCs that may be more relevant in the space environment and also during radiation exposure in the time of cancer treatment. The different biological effects between photon and ^56^Fe irradiation on human bone marrow-MSCs have also been thoroughly characterized *in vitro* (68). While it was reported that neither photon nor ^56^Fe ions impaired the osteogenic differentiation capability of MSCs, ^56^Fe irradiation with 1 Gy resulted in a G2/M-phase arrest, which was more pronounced than after physically equivalent doses of photon irradiation. Microarray analyses showed that several genes playing a role in cell cycle progression including cyclin B1 and cyclin E2 were significantly downregulated after 0.1 Gy ^56^Fe ions, while only a marginal response was observed after photon radiation. Additionally, a more pronounced activation of p53 was found after ^56^Fe ion radiation than after photon treatment.

**Table 1:** Summary of preclinical studies that investigated the effects of different types of particle irradiation on MSCs.

Authors and year	Reference	MSCs’ species and tissue/Animal model	Particle type	Main findings
Almeida-Porada et al., 2018	(66)	Human bone marrow	Protons +^56^Fe	More pronounced deleterious effects after sequential proton and ^56^Fe ion IR on both MSCs and HSCs than after exposure to either ion alone Upregulation of cytokines involved in the maintenance of hematopoiesis and immune cell development after ^56^Fe ion IR (but downregulation after proton IR) Persistence of transcriptional changes induced by protons and ^56^Fe ions over several passages in culture (in contrast to photons)
Alessio et al., 2017	(67)	Human bone marrow	α particles	Reduction of S-phase cells after 0.04 Gy and 2 Gy α particle IR Elevated apoptosis rates at 48 hours after 2 Gy α particle IR but not after photons More residual DNA double-strand breaks at 48 hours after exposure to 2 Gy α particles compared to 2 Gy photons Increased pATM activation after 2 Gy α particle IR than after 2 Gy photon IR
Kurpinski et al., 2009	(68)	Human bone marrow	^56^Fe	Pronounced G2/M phase arrest after 1 Gy^56^Fe IR Maintenance of osteogenic differentiation after 1 Gy^56^Fe IR Higher p53 activation after ^56^Fe exposure compared to photons More pronounced transcriptomic effects regarding DNA replication, DNA strand elongation and DNA binding/transferase activity for^56^Fe than for photons
Nicolay et al., 2015	(69)	Human bone marrow	^12^C	RBE values of ^12^C between 2.0 and 3.1 (at 10% clonogenic survival) Maintenance of stem cell characteristics Pronounced G2/M phase arrest after 4 Gy^12^C IR No increases in apoptosis after 4 Gy^12^C IR No residual DNA double-strand breaks after 4 Gy^12^C IR Strong phosphorylation of pATM at 2 hours after 4 Gy^12^C IR but return to baseline levels after 24 hours

HSC, hematopoietic stem cell, IR, ionizing radiation, RBE, relative biological effectiveness.

While these analyses were based on 2D systems, some groups also investigated the effects of particle irradiation on adipose-derived stem cells in 3D sphere cultures based on agar coating (70). Both after photon and carbon ion radiation, radiation sensitivity was higher in 2D culture than in 3D culture, which could to some extent be related to the development of radioprotective tissue hypoxia inside of the 3D spheres. In this regard, one should take into consideration that particle irradiation can partly overcome the radioresistance caused by tissue hypoxia (71).

### Stem Cell Characteristics After Particle Irradiation

The influence of particle radiation on the defining stem cell characteristics has been investigated both for ^12^C and ^56^Fe ions. After ^12^C irradiation, bone marrow-derived MSCs maintained their adhesive and cellular motility abilities independently of the MSC donor as well as their multi-lineage differentiation capability, which are all pre-requisites for the cells’ regenerative effects (69). Kurpinski et al. could show that the osteogenic differentiation potential of human bone marrow-derived MSCs was maintained after exposure to 1 Gy ^56^Fe ions. While undirected cellular motility as assessed by time-lapse microscopy remained unaffected after ^12^C irradiation, directed migration towards particle-irradiated hematopoietic cells has not been examined so far. For lighter alpha particles, the preservation of MSC functions seems to depend on the dose, and only higher doses (2 Gy) have been shown to inhibit the stemness capacity of bone marrow MSCs (67).

### DNA Damage Repair After Particle Irradiation

As particle irradiation induces clustered and more complex DNA lesions than photon irradiation, many cell lines have been found to exhibit increased numbers of initial and residual DNA lesions after physically equivalent doses of particle radiation (72–74). However, γH2AX foci analyses in human bone marrow-derived MSCs revealed an effective DNA double strand-break repair after 4 Gy ^12^C irradiation as evident by low numbers of residual double strand-breaks and only temporary activation of the DNA double-strand break signaling pathways (69). This effective DNA double strand-break repair may also contribute to the reported low apoptosis rates after ^12^C irradiation in this study (69). However, in the study of Alessio, residual γH2AX levels were significantly higher after 2 Gy alpha particle irradiation than after 2 Gy photon irradiation in bone marrow-isolated MSCs (67). In line with these findings, the authors also observed increased pATM expression at 48 hours after 2 Gy alpha particle irradiation when compared to baseline levels, which was not observed after 2 Gy photon irradiation. Lower alpha particle doses of 0.04 Gy were found to result in an upregulation of pATM at 1 hour after exposure and a decline at later timepoints; additionally, there was no significant difference regarding the number of γH2AX-positive cells at 48 hours after 0.04 Gy alpha particle irradiation compared to unirradiated controls. In the study of Kurpinski and colleagues, pathway and network analyses of transcriptomic profiles revealed differential effects of ^56^Fe and photons on DNA replication, DNA strand elongation and DNA binding/transferase activity of human bone marrow-MSCs with a more pronounced effect of ^56^Fe ions on these pathways. Furthermore 1 Gy^56^Fe ions resulted in a stronger activation of p53 than 1 Gy photons.

### MSC-Based Treatment Concepts for Space Radiation-Induced Toxicities

Galactic cosmic rays (GCR) and solar cosmic radiation (SCR) constitute the main components of space irradiation. With an proportion of about 90%, protons form by far the largest component of both GCR and SCR, followed by helium ions (about 10%), electrons and heavier ions (75). Solar particle events (SPEs) as part of SCR, are unpredictable events that occur when protons are accelerated during a flare or during a coronal mass ejection. Besides highly energetic protons and helium ions, SPEs include heavier charged (HZE) particles such as carbon, oxygen and iron ions. Long-term missions to the Mars that may last more than 2 years harbor the risk for considerable radiation exposures, as the shielding provided by the Earth’s magnetic field is absent. Therefore, the National Aeronautics and Space Administration (NASA) classifies the ARS caused by SPEs as a major obstacle to long-term manned space expeditions (76).

Besides higher doses encountered by SPEs, cumulative annual GCR particle doses inside a space craft amount to 176 ± 29 mGy based on measurements in the Mars Science Laboratory (77). *In vivo* data demonstrated that even low and protracted oxygen ion (^16^O) radiation doses of 0.1 Gy impair hematopoiesis (78), and the effective proton dose to reduce the whole blood cell count (WBC) by half was shown to be approximately 1 Gy (79).

A major step towards the usage of MSCs for space irradiation-induced toxicities was performed recently by Huang and colleagues, who demonstrated the feasibility to grow human bone marrow-MSC aboard the International Space Station (ISS) (80). The MSCs’ phenotype was observed unaltered during proliferation in space, and MSCs maintained their proliferative characteristics aboard ISS. Interestingly, space-expanded MSCs seem to exhibit pronounced immunosuppressive effects compared to MSCs grown on the Earth. At least in short-term space cultures, there were no signs for tumorigenic transformation and genomic instability in MSCs.

Recently, a Chinese study reported transcriptional changes of murine bone marrow cells after whole-body ^12^C irradiation with 2 Gy (81), that would mimic the acute bone marrow exposure during a severe SPE. ^12^C irradiation resulted in increased reactive oxygen species production, γH2AX foci and apoptosis levels in bone marrow cells. Significant alterations in genes belonging to the immune response, DNA damage repair, MAPK, TNF signaling and apoptosis pathways were observed in murine bone marrow cells after 2 Gy ^12^C irradiation.

A high-quality study simulating the combined effects of GCR and SPE on the interaction between human bone marrow MSCs and hematopoietic progenitor cells was conducted by Almeida-Porada (66). Interestingly, ^56^Fe ions upregulated many cytokines involved in hematopoiesis and immune cell development, whereas proton irradiation produced the opposite effect and downregulated key cytokines involved in these functions. Also very importantly, the transcriptional changes after proton and ^56^Fe ion irradiation were long-lasting and persisted over several passages.

It is important to consider that low-dose irradiation (≤ 0.1 Gy) has shown to have significantly different effects on MSCs and adipose-derived stem cells than higher radiation doses (24, 82, 83). Low dose irradiation was found to enhance proliferation and to increase the secretion of stem-cell factor (SCF) and GM-CSF, which may favor hematopoiesis (83). In the study of Yang et al., the pro-survival effects of low-dose irradiation on human bone marrow MSCs were mediated *via* several proteins involved in cell cycle control such as Rb, cyclin E, CDK1, and CDC25B (83). Following these findings, there are considerations to use low-dose irradiation to support large scale expansion as well as therapeutic effects of MSCs (82, 84). Unfortunately, to the best of our knowledge, no studies have reported effects of low-dose particle irradiation, especially protons, on the proliferation rate of MSCs. Additionally, protracted low-dose irradiation over several weeks to mimic GCR are complicated by the difficulty to long-term culture MSCs due to premature senescence and therefore limited passage numbers (85). In the case of prolonged MSC culturing, which may be necessary to expand autologous MSC of astronauts prior to space missions, one should be aware that the DNA repair capacity is lowered leading to more spontaneous and radiation-induced micronuclei (86).

In the space environment, the more convenient way would be the usage of commercially available allogenic off-the-shelf MSCs. Due to the low immunogenicity and immune-privileged behavior, allogenic MSC treatments are generally feasible without prior immune suppression, as demonstrated in large clinical trials (10, 87). In this regard, effective shielding of these off-the-shelf MSCs would be desirable to avoid damage prior to application in a case of SPE.

## Conclusion

In general, MSC-based therapies hold promise for the treatment of irradiation-induced toxicities such as mucositis, osteoradionecrosis, proctitis or cystitis as typical sequelae after radiotherapy in the head-and-neck or pelvic region, respectively. So far, preclinical and clinical research has been conducted focussing on the effects of MSCs on photon-induced toxicities, wherefore the role of MSCs as cell therapy for particle irradiation-related adverse reactions in cancer treatment remains to be elucidated. Furthermore, MSC-based therapies may be used after nuclear accidents or during future manned space missions, although the evidence is very limited. In summary, many further efforts are needed in the future to fully examine the impact of particle irradiation on the regenerative abilities of MSCs and potential attenuating effects of MSCs on particle irradiation-induced normal tissue toxicities.

## Author Contributions

AR, A-LG, and NN wrote and revised this mini-review. All authors contributed to the article and approved the submitted version.

## Funding

AR was supported by the IMM-PACT-Program for Clinician Scientists, Department of Medicine II, Medical Center – University of Freiburg and Faculty of Medicine, University of Freiburg, funded by the Deutsche Forschungsgemeinschaft (DFG, German Research Foundation) – 413517907. We thank the European Space Agency (ESA) for their financial support of the project “Human mesenchymal stem cells as potential mitigators of bone marrow toxicity caused by spaceradiation” within the CORA-IBER program.

## Conflict of Interest

The authors declare that the research was conducted in the absence of any commercial or financial relationships that could be construed as a potential conflict of interest.
